# The Pattern and Characteristics of Childhood Unintentional Injuries in Abha Maternity and Children Hospital, KSA: Prospective Descriptive Study

**DOI:** 10.2196/83867

**Published:** 2026-03-27

**Authors:** Ihab Mohammed Ibrahim Elsorogy, Niemat Mohammed Tahir Ali, Alam Eldin Musa Mustafa, Ibrahim Alhelali, Ibrahim AL-Benhassan, Ahmed Alhijab A Alhazmi, Safa Ahmed Ali Fadoul, Emad Mohammed Mosaad Mohammed, Mohammed Mahmoud Mohammed Almusdi

**Affiliations:** 1 Pediatric Intensive Care Unit, Abha Maternity and Children Hospital, Abha, Saudi Arabia; 2 Department of Child Health, College of Medicine, King Khalid University, P.O. Box 641, Abha, Saudi Arabia, 966 557548475; 3 Department of Pediatrics, Faculty of Medicine, University of Kordofan, Al Ubayyid, Sudan; 4 Pediatric Intensive Care Unit, Khamis Mushait Maternity and Children Hospital, Khamis Mushait, Saudi Arabia

**Keywords:** pattern, characteristics, unintentional injuries, children, complications

## Abstract

**Background:**

In Saudi Arabia, unintentional injuries among children represent a prevalent and significant public health issue and severe injuries are of the leading indications for hospitalization and impairments.

**Objective:**

This study aimed to describe the pattern of unintentional trauma in children admitted to Abha Maternal and Children Hospital, South region of Saudi Arabia.

**Methods:**

This study was a prospective descriptive, cross-sectional, hospital-based study, which was conducted in the Pediatric Intensive Care Unit, Maternity and Children’s Hospital, Abha, Aseer region, Saudi Arabia. This is the central and main hospital in the region but not the only hospital receiving childhood injuries. The study period was from January 2023 to January 2024. Children’s age groups were from 0 to 12 years old. All children in the study were admitted with a diagnosis of unintentional injuries, like RTAs (road traffic accidents), falls, and other home accidents. The study included 104 children and the data collected were analyzed using SPSS (version 27; IBM Corp). Appropriate statistical tests were used for the analysis and all tests were two tailed and probability *P*≤.05 is considered significant.

**Results:**

The sample size of the study was 104 children. The gender distribution was 35 females (33.7 %) and 69 males (66.3 %). The patients were recruited from 18 cities in the Aseer region. About half of the patients (49%) were aged 6‐12 years. Road traffic accidents (RTA) represent the highest percentage of accidents, with 66 (63.5%) children, followed by falls from height with 38 (36.5%) patients. The most significant types of injuries were head and brain injuries 37 (35.6%), followed by chest and lung injuries 12 (11.5%). Most patients (n=62, 59.6%) remained admitted to the pediatric intensive care unit (PICU) for one to three days. Followed by three to seven days (27), then eight to 14 days (14). Head/brain axonal injury is also the most common injury associated with complications, followed by polytrauma.

**Conclusions:**

Road traffic accidents are a significant cause of death and disability in Saudi Arabia for all age groups. A strong association existed between the PICU admission duration and the outcome (*P*=.02). Health and community institutes and governments should increase community education about the risks and consequences of RTA, strengthen traffic rules and laws, and punish violators.

## Introduction

Worldwide unintentional injuries contribute enormously to childhood mortality with nearly 1 million deaths per year in children and adolescents below 18 years [1]. Around 12% of the deaths due to injuries occur in children, the most common causative injuries of death in this age group are: road traffic accidents, drowning, burns, falls, and poisonings. Many of these mortalities are preventable [2]. In children and also the injuries largely contribute to child hood morbidity and can lead to severe occupational malfunctioning or non-functioning and/or social and psychological impairment [3]. There may be background risk in many cases [4]. Unfortunately, there is a paucity of data regarding trauma incidence and prevalence, specifically for Saudi Arabian children [5].

Injuries, especially unintentional injuries, are a leading cause of death among children and adolescents and a severe public health problem worldwide [6,7]. Unintentional injury is a disability that occurs under accidental circumstances [8]. The World Health Organization Report 2002 stated that injuries are the sixth leading cause of morbidity and mortality in childhood [9]. One of the most unintentional injuries in the world is road traffic accidents (RTA), which are the primary cause of unnatural deaths in the world and children, causing a loss of more than 260,000 lives in the 0‐19-year age group annually [10] and a significant effect on the world’s economy [11-13].

Investigations have shown that children and adolescents are most vulnerable to accidents. Due to physiological limitations, the growth process and behavioral characteristics (experience, need to test, explore, adventure, and risky behaviors) provide accident conditions for this group. Along with other environmental factors, for example, parents’ low ecological safety levels and children’s supervision and care have led to more severe and dangerous forms [14]. Every year, millions of children lose their lives because of preventable accidents. Burning, poisoning, falls, and trauma (accidents) are the four leading causes of mortality in children. Accidents represent 50% of all deaths among children, and one in every six children admitted to a children’s hospital emergency department is related to accidents [15]. . Therefore, accidents are one of the leading health problems that continually threaten children’s health [16]. This study was conducted due to limited data about the epidemiology of unintentional injuries among Saudi children and adolescents to increase the knowledge about the characteristics of these injuries for further appropriate intervention and to decrease the burden of such terrible events.

The study aimed to determine the pattern and clinical types of the unintentional injuries and trauma in children aged 0‐12 years admitted to the PICU in Abha, Aseer region in KSA; together with determining of the short-term outcome and utilization need of the health facilities of the hospital including ICU admission and duration of hospital stay in this study group.

## Methods

### Study Design

This study is a prospective descriptive, review with follow up of children admitted with unintentional trauma since admission to the hospital and use of the pediatric intensive care unit database during the period of the study.

### Study Setting

Pediatric Intensive Care Unit, Maternity and Children Hospital, Abha, Aseer region, Saudi Arabia.

### Study Population and Sample

The study sample includes 104 of the children admitted with unintentional trauma to the Pediatric Intensive Care Unit, Maternity and Children Hospital (MCH), Abha. Fifteen to twenty pediatric patients were admitted to the pediatric department monthly due to unintentional injury.

Age group: From 0 to 12 years (because MCH receives pediatric patients up to 12 years)

Study period: January 2023–January 2024

Inclusion criteria: Any pediatric patient admitted with a diagnosis of unintentional injury, such as RTA, pedestrians, and falls

Exclusion criteria: Children with burns, poisoning, drowning, foreign bodies, and strangulation were excluded because these cases usually were admitted to other hospitals

Sample size: The formula used for calculation of sample size is:

n = (Z^2 * p * (1-p)) / d^2

Where:

n=sample size

Z=*z* score corresponding to the desired confidence level (eg, 1.96 for 95% CI)

p=expected prevalence or proportion (use 0.5 if unknown)

d=margin of error (eg, 0.05)

Sampling technique: Prospective review of the department database or Logbooks, as well as review of pediatric patients with a diagnosis of trauma

### Data Analysis

Data were collected on a research data collection sheet and Data management and analysis were performed using SPSS ( version 27.0; IBM Corp). Numerical data were summarized using medians and/or ranges. Categorical data were summarized as numbers and percentages. Estimates of the frequency were done using the numbers and percentages. *χ*^2^ or Fisher tests were used to compare between the independent groups with respect to categorical data, as appropriate. Time to discharge was estimated using the Kaplan and Meier method. It was calculated from date of admission to date of discharge. Differences between the survival curves were assessed with the log-rank test. All tests were two-tailed and *P*≤.05 is considered significant.

### Study Recruitment

This included identifying and sourcing of the study population group according to the criteria of the study.

### Ethical Considerations

The study was conducted in accordance with the Declaration of Helsinki and the research committee of Aseer Institutional Review Board, Directorate of Health Affairs, Aseer Region, Ministry of Health, Saudi Arabia, approved the study (REC 8-3-2023 dated 4/3/2023). Informed consent to participate was obtained from families (verbal approval) before being asked about the child. Privacy and confidentiality of the hospital data used was strictly kept and the data used was deidentified.

## Results

The current study included 104 pediatric patients. Ninety-nine patients were admitted to the PICU, three to the pediatric surgical ward under PICU team care, and two to the ER. More than two-thirds (n=69, 66.3%) of cases were males. Nearly half of the cases were aged 6 to 12 years (n=51, 49%). Most cases were of Saudi nationality (n=96, 92.3%). The patients were recruited from 18 cities in the Aseer region. Abha (n=53, 51%) and Khamis Mushet (n=17, 16.3%) had the highest percentage of patients, respectively. Most cases (n=99, 95.2%) had no past medical history. In contrast, a small proportion of patients had medical conditions, such as factor VIII deficiencies, global developmental delay, preterm birth with congenital heart disease, or nonspecific conditions. Road traffic accidents (RTA) represent the highest percentage of accidents, with 66 children representing 63.5%, followed by falls from height with 38 patients representing 36.5%, and both are notably more frequent in the 6‐12 age group. Meanwhile, home accidents accounted for only 3 cases (2.9%) of all accidents. The most used method for transferring patients from the accident site to the nearest hospital was a red-crescent ambulance 47 (45.2%) patients, followed by a family car 46 (44.2%) patients.

More than one-third of patients arrived at MCH 6‐10 hours after the first presentation 41 (39.4%), and nearly one-third at the first 2 hours 31 (29.8%). More than one-third of cases had head and brain injuries 37 (35.6%), followed by polytrauma (24%). More than half of patients 62 (59.6%) remained admitted to the PICU for [1-3] days, with a median length of stay of about two days, ranging from [1-24] days. Most patients 93 (89.4%) were transferred to the pediatric surgical ward (PSW) from the PICU. Only 6 (5.8%) patients were transferred to another hospital, and 5 (4.8%) patients died in the PICU due to severe injuries, such as multiple fractures and head/brain axonal injury. Most patients (n=89, 85.6%) were discharged from the hospital in improved and stable conditions (Table 1).

**Table 1.** Clinical and hospital-related data.^a^

Variables	Values (N=104)
Time of presentation to MCH^b^ after 1st presentation, n (%)	
First 2 hours	31 (29.8)
2‐5 hours	2 (1.9)
6‐10 hours	41 (39.4)
11‐24 hours	18 (17.3)
24‐72 hours	6 (5.8)
More than 72 hours	6 (5.8)
Diagnosis, n (%)	
Head/brain axonal injury	37 (35.6)
Polytrauma	25 (24)
Fracture skull	15 (14.4)
Chest/lung injury	12 (11.5)
Internal abdominal organ injury	7 (6.7)
Normal CT^c^	8 (7.7)
Needed surgical intervention, n (%)	
No	75 (72.1)
Yes	29 (27.9)
Destination of discharge from the PICU^d^, n (%)	
Pediatric surgical ward	93 (89.4)
Other hospital	6 (5.8)
Died	5 (4.8)
Length of stay in PICU (days), n (%)	
1‐3	62 (59.6)
4‐7	27 (26)
8‐14	15 (14.4)
Outcome, n (%)	
Improved	89 (85.6)
Complications, disabilities, and transfer	10 (9.6)
Died	5 (4.8)
Length of hospital stay (days), median (range)	2 (1-25)

a The percentage of the whole group discharge is taken out of the total number of 104 admitted children; while the percentage of discharge in the subgroups inside the table is taken in relation to the total of the same subgroup (e.g. age group).

b Maternity and Children’s Hospital.

c CT: computed tomography.

d PICU: pediatric intensive care unit.

As shown in Table 2, most deaths (n=4/5, 80%) occur among females , and this was statistically significant (*P*=.04). However, there was no statistically significant difference in mortality concerning age, nationality, presence of past medical history, type of trauma, and diagnosis.

**Table 2.** Mortality concerning different factors.

Variables	Alive (n=99), n (%)	Died (n=5), n (%)	*P* value
Age groups			.82
Less than 1 year	12 (12.1)	1 (20)	
1‐5 years	38 (38.4)	2 (40)	
6‐12 years	49 (49.5)	2 (40)	
Sex			.04
Female	31 (31.3)	4 (80)	
Male	68 (68.7)	1 (20)	
Nationality			≥.99
Saudi	91 (91.9)	5 (100)	
Non-Saudi	8 (8.1)	0 (0)	
Past medical history			≥.99
No	94 (94.9)	5 (100)	NA^a^
Yes	5 (5.1)	0 (0)	
Type of trauma			.65
Road traffic accident	62 (62.6)	4 (80)	
A fall from a height	37 (37.4)	1 (20)	
Diagnosis			.67
Chest/lung injury	12 (12.1)	0 (0)	
Polytrauma	22 (22.2)	3 (60)	
Fracture skull	15 (15.2)	0 (0)	
Head/brain axonal injury	35 (35.4)	2 (40)	
Internal abdominal organ injury	7 (7.1)	0 (0)	
Normal CT^b^	8 (8.1)	0 (0)	

a Not applicable.

b CT: computed tomography.

**Figure 1.**
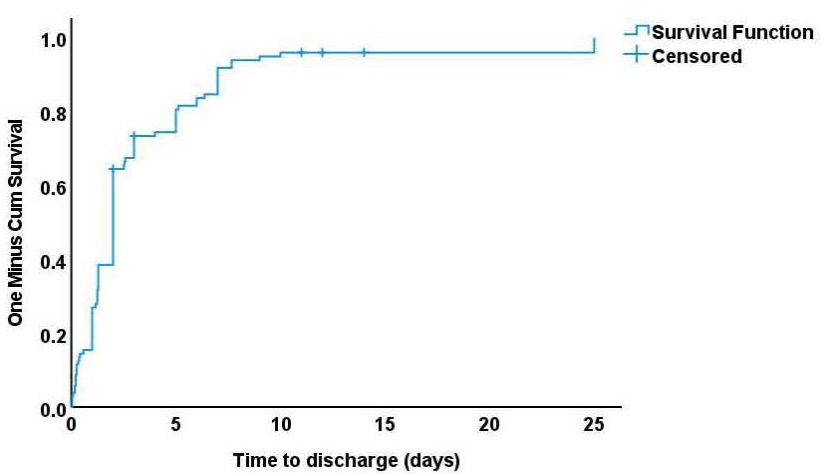
Kaplan and Meier curve representing time to discharge for the whole group.

**Figure 2.**
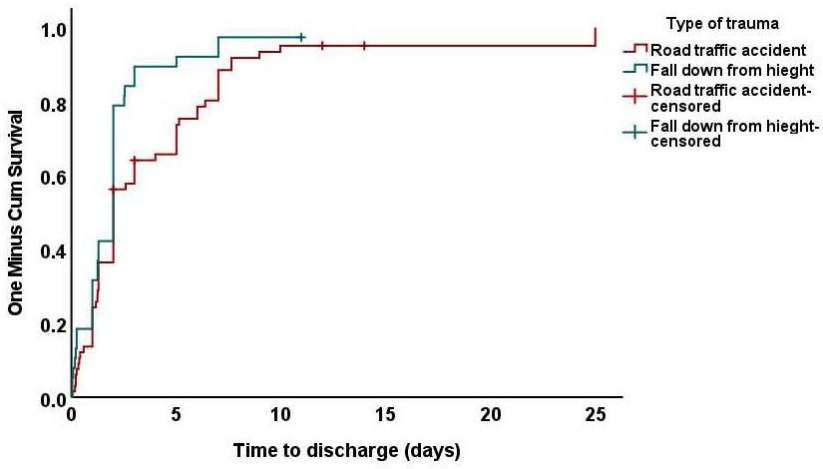
Kaplan and Meier curve representing time to discharge by type of trauma.

As shown in Table 3, the median time to discharge from the hospital was 2 days (Figure 1). The type of trauma significantly impacts time to discharge from the hospital. At one week, 34 out of 37 (93%) of patients who fell from a height were discharged, compared to 50 (81%) of patients who had a RTA. This difference was statistically significant (*P*=.41) (Figure 2). One child who sustained a severe RTA with multiple injuries and disability stayed in the hospital for an exceptional period of 25 days. Meanwhile, there was no statistically significant difference in time to discharge with age, gender, nationality, and diagnosis. The details of the injuries sustained by the children in the study group include the following:

1. Head/brain injuries: 37/104 (35.6%) children, of which 22 (21.2%) were associated with different degrees of brain hemorrhage (epidural, subdural , subarachnoid or intraparenchymal); 6 (5.77%) cases of brain injuries were associated with axonal injury, and 2 (1.9%) cases with fracture base of the skull. Skull fractures were found in 27 (26%) of the cases. Of these, 15 were isolated skull fractures without brain insult and most of them were single bone and nondisplaced with 4 (3.8%) depressed fractures and 3 (2.9%) multiple displaced fractures. A total of 8 cases (7.7%) of head trauma in the group show no abnormal computed tomography or magnetic resonance imaging findings
2. Lung injuries: In 12 (11.5%) cases with chest cage/lung trauma, 2 children (1.9%) had lung contusions, 2 (1.9%) had pneumothorax, 2 (1.9%) cases sustained pulmonary hemorrhage and 1 (0.96%) patient had surgical emphysema
3. Intraabdominal soft tissue organ injuries: 3 cases (2.9%) had splenic lacerations, 2 (1.9%) with liver injury and lacerations, 1 case (0.96%) with renal injury, and 1 case (0.96%) with direct arterial vascular injury
4. Multiple or major limb fractures: i5 cases (4.8%) of polytrauma were seen in the group

**Table 3.** Time to discharge concerning different factors.

Factors	Discharged patients at 1 week	Median time of discharge (days)	*P* value
Whole group, median (range)	85 (86)	2	
Age groups, median (range)			.36
Less than 1 year	11 (93)	2	
1‐5 y	37 (98)	2	
6‐12 y	40 (81)	2	
Sex, median (range)			.90
Female	28 (91)	2	
Male	57 (84)	2	
Nationality, median (range)			.61
Saudi	76 (84)	2	
Non-Saudi	8 (100)	2	
Type of trauma, median (range)			.04
Road traffic accident	50 (81)	2	
A fall from a height	34 (93)	2	
Diagnosis, median (range)			.41
Chest/lung injury	12 (100)	2.5	
Polytrauma	19 (85)	2	
Fracture skull	15 (100)	2	
Head/brain axonal injury	28 (79)	2	
Internal abdominal organ injury	7 (100)	2	
Normal CT^a^	8 (100)	1.3	

a CT: computed tomography.

Most of the admitted children with different types of trauma improved with the treatment; some sustained complications or transferred and 5 died (Table 4).

**Table 4.** Outcome of each diagnosis.

	Chest/lung injury, n=12 (%)	Polytrauma, n=25 (%)	Fracture skull, n=15 (%)	Head/brain axonal injury, n=37 (%)	Internal abdominal organ injury, n=7 (%)	Normal CT, n=8 (%)	*P* value
Outcome							
Improved	11 (91.7)	18 (72)	15 (100)	30 (81.1)	7 (100)	8 (100)	NA^a^
Complications, disabilities, and transfer	1 (8.3)	4 (16)	0 (0)	5 (13.5)	0 (0)	0 (0)	
Died	0 (0)	3 (12)	0 (0)	2 (5.4)	0 (0)	0 (0)	

a NA: Not applicable

The hospital stay was significantly more extended among patients who died at the end compared to patients who improved (*P*=.02) (Table 5).

**Table 5.** Time of arrival to hospital and length of hospital stay and their relation to patient outcome.

Time of arrival to hospital and length of hospital stay	Outcome			*P* value^a^
	Improved (n=89), (%)	Complications, disabilities, and transfer, (n=10), ( %)	Died (n=5), (%)	
Time of presentation to MCH^b^, median (range)				
First 2 hours	27 (30.3)	1 (10)	3 (60)	NA^c^
2‐5 hours	1 (1.1)	1 (10)	0 (0)	
6‐10 hours	34 (38.2)	6 (60)	1 (20)	
11‐24 hours	16 (18)	1 (10)	1 (20)	
24‐72 hours	6 (6.7)	0 (0)	0 (0)	
More than 72 hours	5 (5.6)	1 (10)	0 (0)	
Length of stay in PICU^d^ (days), median (range)				
1‐3	58 (65.2)	4 (40)	0 (0)	NA
4‐7	22 (24.7)	3 (30)	2 (40)	
8‐14	9 (10.1)	3 (30)	3 (60)	
Length of hospital stay (days), median (range)	2 (0‐10)	2 (0.3‐25)	11 (2‐14)	.03

a *P*<.05 is considered significant.

b MCH: Maternity and Children’s Hospital.

c NA: Not applicable.

d PICU: pediatric intensive care unit.

## Discussion

### Principal Findings

Unintentional injuries are accidents or mishaps that cause physical harm to a person. These can include falls, traffic accidents, burns, poisonings, drowning, and various other incidents. Over 900,000 deaths are reported annually due to unintentional injuries in the group of children and adolescents below the age of 18 years , representing 10% of all deaths worldwide [17].

The major findings in this study: of the 104 children with unintentional injuries admitted to Pediatric Intensive Care Unit, MCH, Abha, were as follows: A total of 69 (66.3 %) of patients were males, about half of the patients (n=51, 49%) were aged 6‐12 years. Road traffic accidents (RTA) represent the highest percentage of accidents, with 66 (63.5%) children, followed by falls from height with 38 (36.5%) patients, The most significant types of injuries were head and brain injuries (n=37, 35.6%), followed by chest and lung injuries (n=27, 26.1%). Most patients (n=62, 59.6%) remained admitted to the PICU for one to three days. Followed by three to seven days in 27 patients (26%) then eight to 14 days in 15(14.1%). Head/brain axonal injury is also the most common injury associated with complications, followed by polytrauma. Overall, 89 (85.6%) patients improved and discharged with no sequelae, 10 (9.6%) were transferred to other units or develop disabilities and 5 patients (4.8%) died.

This study included 104 pediatric patients. Males dominated, accounting for 69 (66.3%) of the study population. This percentage is similar to that reported in studies conducted in Saudi Arabia [16,18,19]. Although there is male predominance, the rate of death was significantly higher (*P* value =.043) in females. Four female patients from the 31 admitted to the PICU died. Most patients were 6‐12 years old, unlike in previous studies in China and the Netherlands, where age groups 1‐3 predominated [20,21]. .

The commonly admitted age group in Saudi Arabia was 1‐5 years [18,19]. This variation in our study can be attributed to RTA, the most common cause of unintentional injuries (representing 66.3% of the study population), followed by falls from heights. RTA has a high incidence in Saudi Arabia, with 81 percent of deaths due to road traffic accidents in the Ministry of Health hospitals, and 20% of the hospital beds occupied by traffic accident victims [22]. Falls represented a higher percentage in similar studies where the smaller age group dominated [16,18]. There is variation in the type of injury according to age group, where RTA predominates in the 6 to 12-year age group, while falling is more common in the younger age groups of 1-to 5-year-olds and less than one year. A study conducted in Makkah explained the higher incidence of falling as a cause of unintentional injuries in their research because of the predominance of the younger age group from one to five years. It attributed this to this age group’s curious nature and immature judgment [18]. Another study stated that the incidence of falling decreases with increasing age [23]. The mechanism of injury can differ in different societies. Foreign body aspiration and suffocation were the most common admission causes leading to death in China, where RTA came in third place [17,20]. The incidence and type of unintentional trauma can be attributed to environmental factors, parental neglect, or maltreatment [17,24]. The most used method for transferring patients from the accident site to the nearest hospital was a red-crescent ambulance 47 (45.2%), followed by a family car 46 (44.2%). More than one-third of patients arrived at MCH 6‐10 hours after the first presentation 41 (39.4%), and nearly one-third at the first 2 hours 31 (29.8%). Eighty-nine of patients (85.6%)were discharged from the hospital in an improved and stable condition. The mortality rate among the patients was comparatively low 5 patients (4.8%). This suggests that although some individuals exhibited injuries that were too grave to survive, the mortality rate remained regulated, possibly indicative of adequate medical care in most cases. The outcome was also good in other studies in Saudi Arabia, where the majority improved; however, the percentage of discharge with complications and disabilities was higher in different studies [18,19]. . The incidence of death was higher in patients with a longer duration of admission to the PICU. A strong association existed between the PICU admission duration and the outcome (*P*=.023).

The most significant types of injuries were head and brain injuries 37 (35.6 %), followed by polytrauma 22 (21.2%). Head and brain injuries and polytrauma carry a higher percentage of disabilities, complications, longer duration of admission, and death than other diagnoses. This result agrees with similar studies in Saudi Arabia [16,18] Similar results were also found in other countries [24].

Road traffic accidents are a significant cause of death and disability in Saudi Arabia for all age groups. Health and community institutes and governments should increase community education about the risks and consequences of RTA, strengthen traffic rules and laws, and punish violators. Expanding road safety measures to prevent head injuries is also important.

### Limitations of Discussion

For more generalizations of the results and implications of this study; further elaboration of the problem on a wider basis is needed for this area of KSA to include all the hospitals in the region of Aseer

### Conclusion and Implications

Many cases of the unintentional trauma in children in Aseer region in KSA are preventable by using measures to raise the population safety of the RTA as they represent a major type of injury in this region and also safety measures for decreasing the incidence of fall especially the area is a high altitude area in KSA.

## Abbreviations

MCH: Maternity and Children Hospital
PICU: pediatric intensive care unit
RTA: road traffic accident
